# A Search for Dual Action HIV-1 Reverse Transcriptase, Bacterial RNA Polymerase Inhibitors

**DOI:** 10.3390/molecules22111808

**Published:** 2017-10-25

**Authors:** Agata Paneth, Tomasz Frączek, Agnieszka Grzegorczyk, Dominika Janowska, Anna Malm, Piotr Paneth

**Affiliations:** 1 Department of Organic Chemistry, Medical University of Lublin, 20-093 Lublin, Poland; dominika.hagel@gmail.com; 2 Institute of Applied Radiation Chemistry, Lodz University of Technology, 90-924 Lodz, Poland; tomasz.fraczek@p.lodz.pl; 3 Department of Pharmaceutical Microbiology, Medical University of Lublin, 20-093 Lublin, Poland; agnieszka.grzegorczyk@umlub.pl (A.G.); anna.malm@umlub.pl (A.M.)

**Keywords:** triazoles, antibacterial activity, molecular modeling

## Abstract

Using molecular modeling approach, potential antibacterial agents with triazole core were proposed. A moderate to weak level of antibacterial activity in most of the compounds have been observed, with best minimal inhibitory concentration (MIC) value of 0.003 mg/mL, as shown by the **15** against *S. epidermidis.* Studied compounds were also submitted to the antifungal assay. The best antifungal activity was detected for **16** with MIC at 0.125 and 0.25 mg/mL against *C. albicans* and *C. parapsilosis*, respectively.

## 1. Introduction

**Figure 1:**
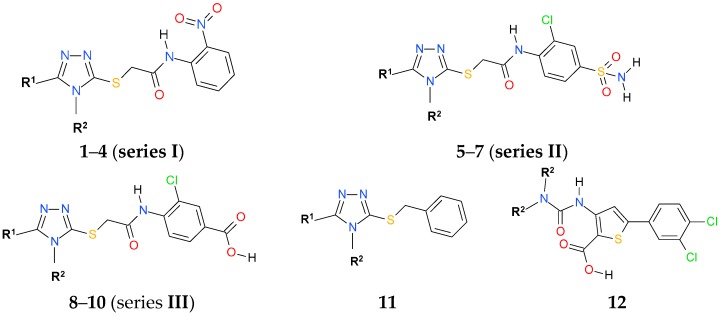
General structures of our recently reported triazole non-nucleoside reverse-transcriptase inhibitors (NNRTIs) [6] and thiophene-2-carboxylic acid derivative (**12**) with inhibitory activity against RNA polymerase (RNAP) [14].

Human immunodeficiency virus infection is associated with the progressive loss of CD4+ T cells and massive dysregulation of the immune system [1]. Such conditions, commonly referred to as acquired immunodeficiency syndrome (AIDS), or AIDS related complex (ARC), make HIV patients vulnerable to opportunistic infections such as bacterial, fungal, viral, protozoal, and neoplastic diseases, which ultimately lead to death. Indeed, according to global HIV and AIDS statistics, since the start of the epidemic, an estimated 78 million people have become infected with HIV and 35 million people have died of AIDS-related illnesses [2]. Despite the recent success of highly active antiretroviral therapy (HAART) in improving the prognosis for HIV-infected individuals, challenges to effective use of these therapeutic strategies remain, including issues of adherence, toxicities, detrimental side effects, drug resistance, and persistent viral replication in latent reservoirs [3]. Treatment of bacterial/HIV-1 co-infection is even more challenging due to potential drug-drug interactions and the ongoing prevalence of resistant strains in HIV-1 populations [4]. Therefore, there is an urgent need to develop anti-HIV agents with dual antiviral and antibacterial potency. The simplification of antiretroviral therapy to single-pill combination would not only offer the convenience to address the above mentioned issues but it would also contribute to improving patient satisfaction, their quality of life, medication adherence, and lowering the health care costs [5]. In this scenario, our recently reported triazole non-nucleoside reverse-transcriptase inhibitors (NNRTIs) [6] (Figure 1) have emerged as very suitable pharmacophore structures. Results revealed that the most potent of the studied compounds had IC_50_ in the range of 3.3–18.5 µM against the HIV-1 wild-type, making them a good starting point for developing antiretroviral drugs. In turn, bacterial RNA polymerase (RNAP), a proven target for broad-spectrum antibacterial therapy [7,8,9], is structurally and functionally similar to reverse transcriptase (RT) [10]. Consequently, it is reasonable to suppose that our NNRTIs should also inhibit RNAP. We have therefore submitted compounds to molecular docking studies in the RNAP “switch region” with a mechanistic function close to that of the NNRTI binding site that is followed by antimicrobial assay. The results of these combined in silico and experimental studies are presented in this paper.

## 2. Results and Discussion

### 2.1. Rationale

As mentioned in the Introduction, the “switch region” has been discovered as an interesting new binding domain of bacterial RNAP. A natural antibiotic myxopyronin B and its synthetic derivative desmethyl myxopyronin B have been demonstrated to target this region [11]. Although myxopyronin B is highly active in vitro, its clinical use is hampered by insufficient physicochemical properties [12,13]. Therefore, novel RNAP “switch region” inhibitors are being developed; due to their different binding mode as compared to rifamycin antibiotics, such inhibitors are expected to overcome existent resistance [9].

Based on the functional and structural similarities between bacterial RNAP “switch region” and the NNRTI allosteric binding site, following a structure-based design strategy, Hartmann et al. identified the first synthetic 2-ureidothiophene-3-carboxylic acids as dual RNAP/RT inhibitors [14]. The compounds were characterized by a potent antibacterial activity against *S. aureus* and in cellulo antiretroviral activity against NNRTI-resistant strains. Recently, we have identified novel triazole NNRTIs also following the molecular approach. In vitro activity determination using HIV-1 RT wild type inhibition assay led to the identification of the compounds **1**–**11** (Figure 1) showing inhibitory activity with an IC_50_ values in the range of 3.3 to 76 µM. 

To find out whether our previously reported triazole NNRTIs are also good candidates for bacterial RNAP inhibitors, the compounds **1**–**11** were docked to the “switch region” of the RNAP using a cocrystal structure of *Thermus thermophilus* RNAP complex (PDB code 3DXJ) and their bonding mode was analysed. 

**Figure 2:**
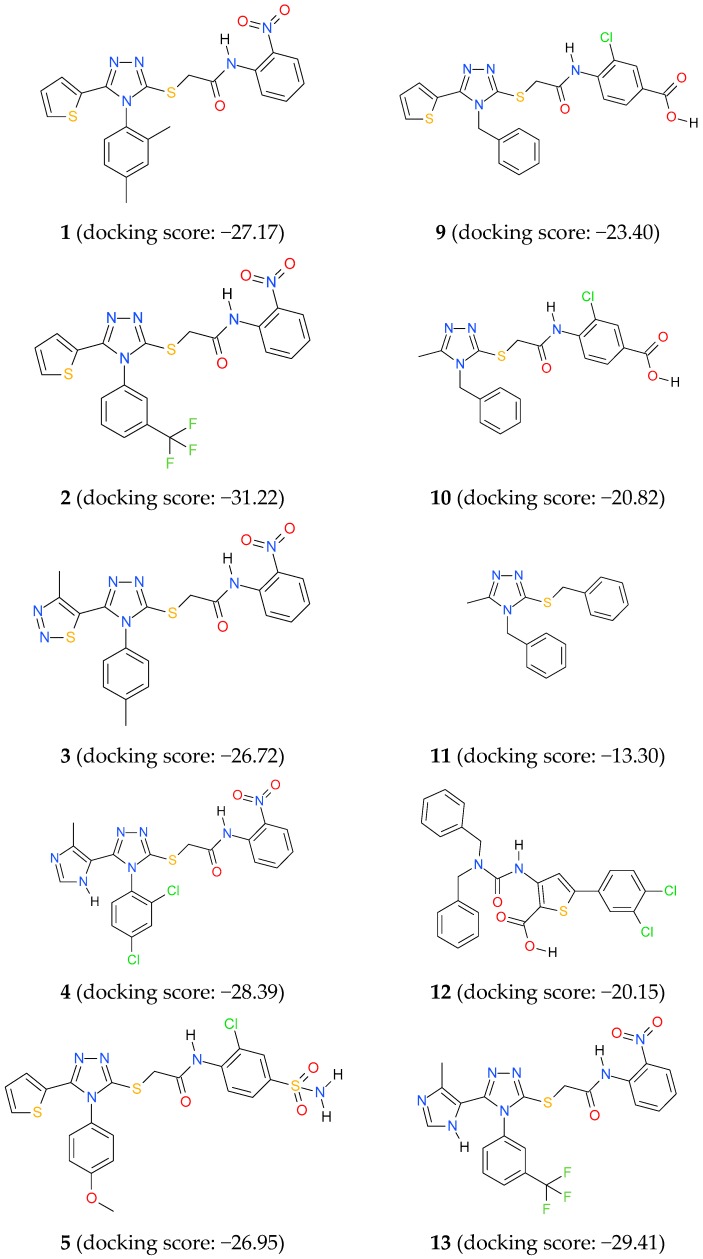

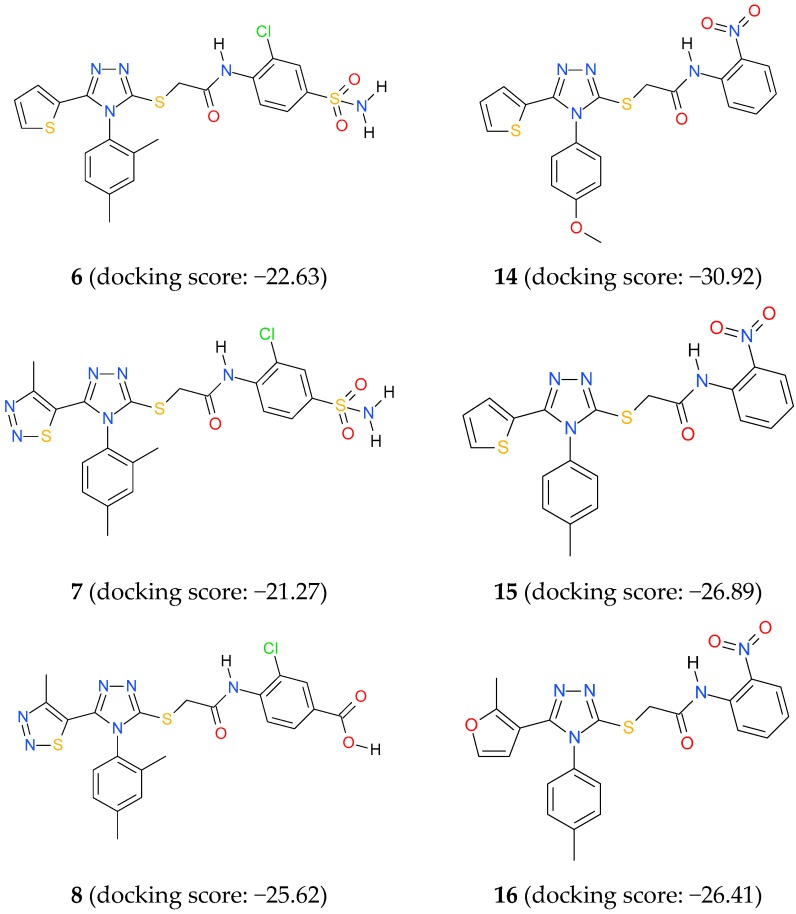
Docking scores for previously reported triazole NNRTIs (**1**–**11**) [6], known bacterial RNA polymerase inhibitor (**12**) [14], and designed triazoles with the *N*-(2-nitrophenyl)acetamide core (**13**–**16**) within bacterial RNAP “switch region”.

**Figure 3:**
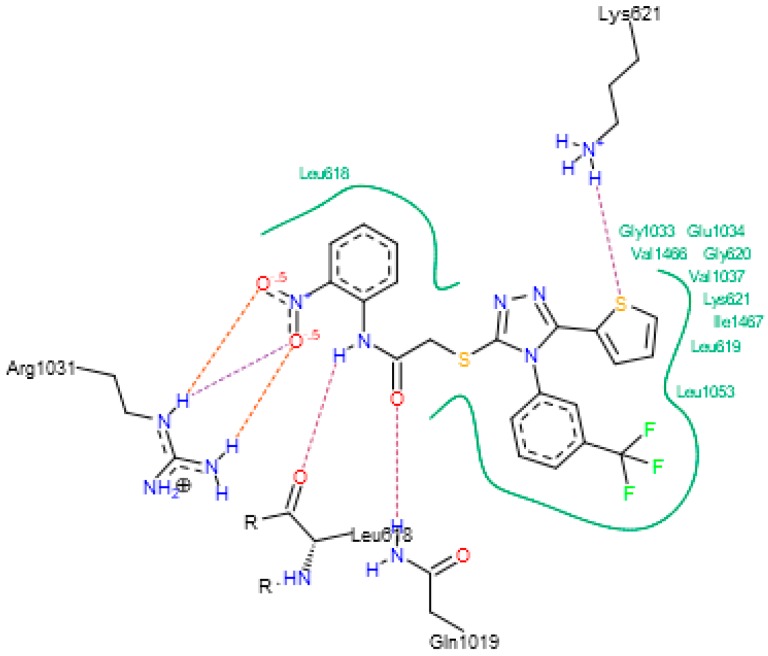
Binding mode of **2** within bacterial RNAP “switch region”.

According to the docking scores presented in Figure 2, with the exception of **11** (docking score −13.30)**,** all of the triazoles series I–III are predicted to bind with comparable or even a higher affinity than both native myxopyronin (docking score −21.29) and previously reported RNAP inhibitor **12** (docking score −20.15). Out of these, the best binding affinity is predicted for triazoles series I with docking score −31.22 (**2**), followed by −28.39 (**4**), −27.17 (**1**), and −26.72 (**3**) due to their ability to form intermolecular hydrogen bonds in the nitrophenyl-amide core. Indeed, as depicted in Figure 3, for the compound **2** with the best docking score, its position in the binding pocket is stabilized by three H-bond interactions of its nitro group with the amine groups of Arg1031 and two H-bond interactions (as donor and acceptor) of its amide group with the C=O group of Leu618 and the H-N group of Gln1019. Its binding is further strengthened through close interactions of the thiophene ring with Gly1033, Glu1034, Val1466, Gly620, Val1037, Lys621, Ile1467, Leu619, and Leu1053.

**Figure 4:**
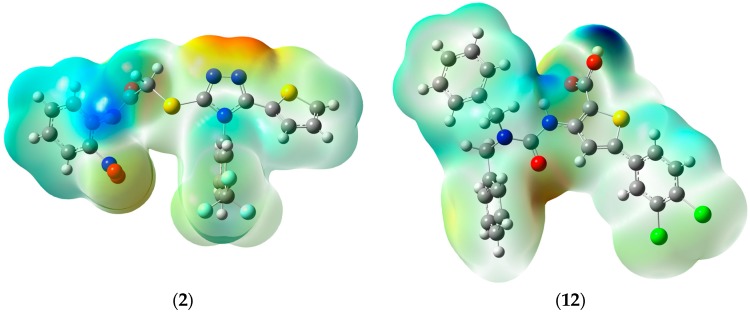
Molecular electrostatic potential (MEP) of **2** and **12**.

Since the final recognition in binding site is likely to be dominated by electrostatic forces [15], geometry optimizations of **2** and previously reported RNAP inhibitor **12** were carried out and molecular electrostatic potentials (MEP) were visualized (Figure 4). Neutral fragments are characterized by green color, the negative charge is represented by red, while the positive charge is marked in blue. As can be seen, both of the compounds seem to be electrostatically similar enough to be recognized by the enzyme.

To prove the favoured binding mode in the “switch region” of the bacterial RNAP proposed for triazoles with the *N*-(2-nitrophenyl)acetamide core four potential inhibitors **13**–**16** with higher affinity than native myxopyronin were designed (see Figure 2), and their antibacterial potency against gram-positive and gram-negative bacterial strains was tested in vitro. For antibacterial assay, two previously reported triazoles series I with the best docking scores, i.e., **2** and **4**, were also selected.

### 2.2. Chemistry

**Scheme 1:**
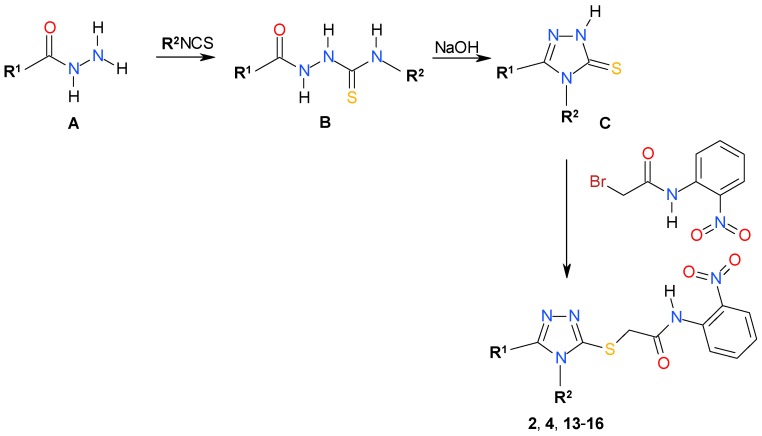
Synthetic route for triazoles (**2**, **4**, **13**–**16**).

Synthesis of triazoles series A (**2**, **4**, **13**–**16**) was accomplished through a three-step procedure starting with appropriate carboxylic acid hydrazide **A** (Scheme 1). Reaction with isothiocyanate followed by basic cyclization of the produced thiosemicarbazides **B** delivered the intermediate triazoles **C** in a good yield. Reaction of triazoles **C** with 2-bromo-*N*-(2-nitrophenyl)acetamide in the presence of potassium iodide or tetrabutylammonium iodide and potassium carbonate yielded the final triazoles (**2**, **4**, **13**–**16**) in a one-pot reaction.

### 2.3. Antimicrobial Assay

The triazoles (**2**, **4**, **13**–**16**) were then submitted for antibacterial assay against a panel of Gram-positive and Gram-negative reference bacterial strains using broth microdilution assay. The ciprofloxacin was included as a control. Results revealed that with the exception of **15** with MIC at 0.003 mg/mL against *S. epidermidis*, all of the tested compounds possessed limited antibacterial activity. Among them, the best inhibitory activity against Gram-positive bacterial strains was observed for **13** (MIC range 0.06–0.5 mg/mL), followed by **15** (MIC range 0.003–1 mg/mL), **16** (MIC range 0.25–1 mg/mL) and almost inactive compounds **2**, **4**, **14**. In all of the cases, MICs for Gram-negative bacteria were higher in comparison to MICs for Gram-positive bacteria, which suggests their lower sensitivity to tested triazoles. Indeed, with the exception of *B. bronchiseptica*, all of the Gram-negative strains were able to grow at high concentration (MICs of 1 mg/mL) or even were insensitive to compounds tested.

Finally, we completed our antibacterial assay by evaluating the antifungal potency of **2**, **4**, **13**–**16** against the yeast *Candida albicans* and *Candida parapsilosis.* The fluconazole was included as a control. No appreciable antifungal activity was observed. As indicated by results collected in Table 1, the best inhibitory activity was observed for **16** with MICs at 0.125 and 0.25 mg/mL against *Candida albicans* and *Candida parapsilosis*, respectively, while no marked difference was observed in the activity profiles for remaining compounds.

## 3. Materials and Methods

### 3.1. Chemistry

All of the reagents and solvents of analytical grade or higher were purchased from commercial sources and were used without purification unless otherwise stated. NMR spectra were recorded using Bruker Avance spectrometers (250, 300, and 700 MHz) (Bruker BioSpin GmbH, Rheinstetten, Germany). Analytical thin layer chromatography was performed with Merck60F254 silica gel plates (Merck, Darmstadt, Germany) and visualized by UV irradiation (254 nm). Melting points were determined on a Fisher–Johns block (Thermo Fisher Scientific, Schwerte, Germany), the reported values are uncorrected. Elemental analyses were determined by an AMZ-CHX elemental analyzer (PG, Gdańsk, Poland) (all results were within ±0.5% of the theoretical values). Mass spectra (ESI TOF) were recorded using Waters LCT Premier XE mass spectrometer (Waters, Milford, MA, USA).

#### 3.1.1. General Procedure for Synthesis of Thiosemicarbazides **B**

A solution of corresponding carboxylic acid hydrazide **A** (0.01 mol) and equimolar amount of appropriate isothiocyanate in 25 mL of anhydrous ethanol was heated under reflux for 2 h. Next, the solution was cooled and the solid formed was filtered off, washed with diethyl ether, dried, and crystallized from ethanol.

#### 3.1.2. General Procedure for Synthesis of Triazoles **C**

A solution of the corresponding thiosemicarbazide derivative **B** (0.01 mol) in 2% sodium hydroxide (10 mL) was refluxed and the progress of the reaction was monitored by thin layer chromatography. After 2 h, the reaction was completed and the reaction mixture was cooled and then acidified with 3 M hydrochloric acid. The precipitate was filtered, washed with water, and crystallized from ethanol.

#### 3.1.3. Synthesis of 2-Bromo-*N*-(2-nitrophenyl)acetamide

*o*-Nitroaniline (0.01 mol) was dissolved in methylene chloride and cooled down to 0 °C. Potassium carbonate (0.03 mol) was added and then freshly distilled bromoacetyl bromide (0.015 mol) was added dropwise for 1–3 min. After the reaction was completed (controlled by TLC, chloroform–methanol 29:1), the precipitate was centrifuged, washed with methylene chloride and discarded. Combined extracts were concentrated under reduced pressure and the product was precipitated with heptane, centrifuged, washed twice with pentane, and dried. 

#### 3.1.4. General Procedure for Synthesis of the Triazoles (**2**, **4**, **13**–**16**)

One equivalent of the corresponding triazole **C** and 1.7 mg of potassium iodide (or tetrabutylammonium iodide, 0.3 eq.) were dissolved/suspended in 2 mL of methanol. 5.2 mg of K_2_CO_3_ (1.1 eq.) was added and the mixture was heated to 60 °C under reflux for 20 min. After cooling to <30 °C, 1.2 equivalent of the 2-bromo-*N*-(2-nitrophenyl)acetamide was added and the reaction mixture was stirred for 1–2 h, monitored by TLC. Subsequently, the reaction mixture was filtered, evaporated, dissolved in methylene chloride, filtered, concentrated, and layered with heptane. The product precipitated or crystallized within 24 h was washed with heptane and dried. If the product separated as an oil, addition of 1–2 drops of methylene chloride often caused solidification.

Physicochemical characterization of all compounds was reported elsewhere [6,16,17]. The data obtained are in agreement with those previously reported.

### 3.2. Antimicrobial Assay

The in vitro antimicrobial activity of (**2**, **4**, **13**–**16**) was tested against a panel of microorganisms from American Type Culture Collection (ATCC): Gram-positive bacteria (*Staphylococcus aureus* ATCC 6538, *Staphylococcus aureus* ATCC 43300, *Staphylococcus aureus* ATCC 25923, *Staphylococcus epidermidis* ATCC 12228, *Micrococcus luteus* ATCC 10240, *Bacillus subtilis* ATCC 6633, *Bacillus cereus* ATCC 10876), Gram-negative bacteria (*Escherichia coli* ATCC 3521, *Escherichia coli* ATCC 25922, *Salmonella typhimurium* ATCC 14028, *Klebsiella pneumoniae* ATCC 13883, *Pseudomonas aeruginosa* ATCC 9027, *Proteus mirabilis* ATCC 12453), *Bordetella brochiseptica* ATCC 4617, and yeasts (*Candida parapsilosis* ATCC 22019, *Candida albicans* ATCC 2091, *Candida albicans* ATCC 10231). Microorganisms were stored in the broth media containing 17% (*v*/*v*) glicerol at −70 °C. Before the experiments, all of the strains were transferred onto Tripticase Soy Agar (in case of bacteria) and Sabouraud agar (in case of yeasts) and incubated in ambient air for 24 h at 35 °C or 48 h at 30 °C for bacteria and yeasts, respectively.

The in vitro antimicrobial activity were performed using broth microdilution method in 96-well microtitrate plates, allowing for estimation of MIC (minimum inhibitory concentration) and MBC (minimum bactericidal concentration) or MFC (minimum fungicidal concentration). Mueller-Hinton broth (MHB) and Mueller-Hinton broth with 2% glucose (MHB 2%) were used to determine antibacterial and antifungal activity, respectively. The tested compounds were dissolved in dimethyl sulfoxide (DMSO) to a concentration of 10 g/L. First, stock solution (1 g/L) of the tested compounds were prepared in MHB and MHB 2%. Then, serial twofold dilution of these compounds in the same media were made in order to obtained final concentrations of samples ranging from 0.008 × 10^−3^ to 1 mg mL^−1^.

The colonies of each strain were resuspended in sterile physiological saline to get an optical density equal to 0.5 McFarland. Then, 2 µL of the suspension was put into the wells to obtained final concentration of inoculum approximately of 106 CFU mL^−1^ and 104 CFU mL^−1^ in case of bacteria and yeasts, respectively. The last two wells were positive and negative controls. The positive control was inoculated with bacteria and yeasts suspension only, while the negative control was left blank without inoculation. The MICs of the compounds were recorded as the lowest concentration where no viability was observed after incubation at 35 °C (in case bacteria) and 30 °C (in case yeasts) in ambient air for 24 h. The results of MICs were checked by the spectrophotometry of absorbance at 580 nm using Absorbance Microplate Reader EL×800 (BioTek Instruments, Inc., Winooski, VT, USA). After determination of the MICs, MBCs, and MFCs were determined by spreading 5 μL suspension from each well showing no growth onto Mueller-Hinton Agar (MHA) for bacteria, and Mueller-Hinton Agar with 2% glucose (MHA 2%) for yeasts. The MBCs and MFCs of the compounds were recorded as the lowest concentration that kills 99.9% of the bacteria (fungi) [18,19]. All of the experiments were performed in triplicate. 

### 3.3. Computational Details

Conformational search was performed using the Amber force field as implemented in HyperChem 8.0.3. [20] and default convergence criteria. For the most stable conformers population analysis was carried out using the Merz-Kollman scheme [21] at the HF/6-31G theory level with the use of the Gaussian package [22].

### 3.4. Docking Studies

The docking simulations were performed using the FlexX docking module of the LeadIT environment as implemented in the LeadIT 2.1.9 program (BioSolveIT GmbH, Augustin, Germany) using cocrystal structure of *Thermusthermophilus* RNAP complex (PDB code 3DXJ). The active sites were defined to include all of the atoms within 10 Å radius of the native ligand. To validate the docking protocol, ligands co-crystallized with the proteins were initially docked into the crystal structure of the appropriate enzymes; the best conformations obtained were practically identical with the experimental ones. Subsequently, studied compounds were docked using the same docking parameters. The first 100 top ranked docking poses were saved for each docking run.

## 4. Conclusions

In this study, molecular docking approach combined with antimicrobial assay was used to find new compounds with dual antiretroviral and antibacterial potency. No appreciable antibacterial activity was found for all of the tested triazole NNRTIs. These results, though seemingly disappointing, could be useful for future research.

## Figures and Tables

**Table 1:** Antimicrobial data (minimal inhibitory concentration (MIC) and minimum bactericidal concentration (MBC), mg/mL) for **2**, **4**, **13**–**16**.

	2	4	13	14	15	16	Control *
**Gram-Positive Bacteria**	**MIC; MBC (mg/mL)**						
*S. aureus* ATCC 6538	1; >1	n.a.	0.5; 2	n.a.	0.5; >1	1; >1	0.24; 0.24
*S. aureus* ATCC 43300	n.a.	1; >1	0.25; 1	n.a.	1; >1	0.25; >1	0.24; 0.24
*S. aureus* ATCC 25923	n.a.	n.a.	0.25; >1	0.5; >1	0.5; >1	0.5; >1	0.49; 0.49
*S. epidermidis* ATCC 12228	1; >1	1; >1	0.06; 0.125	0.5; 2	0.003; 1	0.5; >1	0.49; 0.49
*B. subtilis* ATCC 6633	1; >1	0.125; 1	0.125; 1	1; >1	1; >1	0.25; >1	0.03; 012
*B. cereus* ATCC 10876	1; >1	1; >1	0.5; >1	1; >1	0.5; >1	1; >1	0.12; 0.12
*M. luteus* ATCC 10240	1; 2	1; >1	0.125; 0.5	1; >1	1; >1	0.5; >1	0.98; 1.95
**Gram-negative bacteria**	**MIC; MBC (mg/mL)**						
*E. coli* ATCC 35218	1; >1	n.a.	1; >1	1; >1	n.a.	1; >1	0.015; 0.015
*E. coli* ATCC 25922	1; >1	n.a.	1; >1	1; >1	n.a.	1; >1	0.008; 0.008
*S. typhimurium* ATCC 14028	1; >1	n.a.	1; >1	1; >1	n.a.	1; >1	0.008; 0.008
*K. pneumoniae* ATCC 13883	n.a.	n.a.	1; >1	n.a.	n.a.	1; >1	0.015; 0.015
*P. mirabilis* ATCC 12453	n.a.	n.a.	1; >1	n.a.	n.a.	n.a.	0.015; 0.015
*P. aeruginosa* ATCC 9027	1; >1	1; >1	1; >1	1; >1	1; >1	1; >1	0.12; 0.24
*B. bronchiseptica* ATCC 4617	1; >1	0.5; >1	0.5; >1	0.5; >1	0.5; >1	0.5; >1	0.12; 0.12
**Yeasts**	**MIC; MBC (mg/mL)**						
*C. parapsilosis* ATCC 22019	0.5; 1	0.5; 1	0.5; 1	0.5; 1	0.5; 1	0.25; 1	1.95; 1.95
*C. albicans* ATCC 2091	0.5; 1	0.5; 0.5	0.5; 0.5	0.5; 1	0.5; 0.5	0.125; 0.25	0.98; 0.98
*C. albicans* ATCC 10231	1; 1	1; 1	0.5; 1	1; 1	0.5; 1	1; 1	0.98; 1.95

* MICs (×10^−3^) for ciprofloxacin (antibacterial assay) and fluconazole (antifungal assay).
